# Supplementary material for: CQ-CNN: A lightweight hybrid classical–quantum convolutional neural network for Alzheimer’s disease detection using 3D structural brain MRI
Source: PLoS One. 2025 Sep 22;20(9):e0331870. doi: 10.1371/journal.pone.0331870 (PMC12453261; doi:10.1371/journal.pone.0331870)
Supplement: Attachment — Submitted filename: Response to Reviewers.pdf [file pone.0331870.s001.pdf]

July 19, 2025

Editor-in-Chief
PLOS ONE

Dear Editor-In-Chief,

We want to express our sincere gratitude for granting us the opportunity to submit the revised manuscript (Ref. No.: PONE-D-25-31817) titled "CQ-CNN: A lightweight hybrid classical-quantum neural network for Alzheimer's disease detection using clinical 3D MRI" to PLOS ONE. We greatly value the time and effort you and the reviewers invested in our manuscript, which has significantly improved its quality and rigor.

Your feedback and the reviewers' suggestions were truly invaluable. We are pleased to inform you that we have carefully addressed all the reviewers' questions and comments, making the necessary revisions to the manuscript.

Our responses to the reviewers' comments are included in the following pages, with page numbers referring to the revised manuscript that incorporates tracked changes. As the corresponding author, I confirm that all authors have reviewed the manuscript, agreed to the changes, and consented to the submission of this revised version.

We genuinely appreciate your consideration of our manuscript.
Best Regards,

Dr. M. R. C. Mahdy (Corresponding Author)
Associate Professor
Department of Electrical and Computer Engineering
North South University, Dhaka, Bangladesh
E-mail: mahdy.chowdhury@northsouth.edu

Response to The Editorial Board

Academic Editor's Comments

1. Please ensure that your manuscript meets PLOS ONE's style requirements, including those for file naming. The PLOS ONE style templates can be found at https://journals.plos.org/plosone/s/file?id=wjVg/PLOSOne_formatting_sample_main_body.pdf and https://journals.plos.org/plosone/s/file?id=ba62/PLOSOne_formatting_sample_title_authors_affiliations.pdf

   Response:

   Thank you for your guidance. We have carefully reviewed the PLOS ONE style requirements and ensured that our manuscript now adheres to all formatting and file-naming guidelines. The necessary revisions have been made in accordance with the provided templates.

2. Although reviewers suggested Rejection among some Major Revisions, I think the manuscript merits reconsideration upon satisfactory revisions. Kindly see the comments enclosed. Also, please justify by yourself whether the Ref recommendations, if any, are necessary. Please cite those only if they do improve the manuscript.

   Response:

   We appreciate the editorial decision to offer reconsideration pending satisfactory revision. We have carefully reviewed all reviewer comments and reference suggestions and evaluated their relevance to our framework and empirical findings. Where the recommended citations substantively strengthened methodological clarity, contextual alignment, or discussion depth, we have incorporated them into the revised manuscript with appropriate justification. In cases where the suggested references did not directly support the scope or contributions of our work, we opted not to include them, in alignment with the editorial guidance to maintain citation integrity. A point-by-point response to all reviewer comments and reference suggestions is included in the rebuttal document.

Note to The Reviewers

1. All modified portions in the revised manuscript are highlighted in yellow colored text.
2. In answering the comments, we have used the following format throughout this response letter. We first state the comments made by the respective reviewer, followed by our response.

[**Tribute**] We would like to express our deepest appreciation to the anonymous reviewers for their extremely insightful comments that greatly enhanced the quality of the paper. We have made every effort to implement the suggestions, demonstrating the effectiveness of our work.

Response to The Reviewers

Responses to the review comments are illustrated below:

Reviewer 1

Comment 1.1 – Keep the introduction section concise. Many irrelevant details make it overly lengthy. Focus on explaining the shortcomings of machine learning models in AD, how your strategy addresses these major deficiencies, and why these deficiencies are significant.

Response:

Thank you for the recommendation. Following your suggestion, we rewrote the "Introduction" section, removed all redundant and repetitive passages, and reintroduced the problem statement along with the goal of our research, which is to evaluate the performance of hybrid classical-quantum neural networks for AD detection, where class images are distinctly similar. We also added several prior works on hybrid classical-quantum approaches implemented on quantum hardware and justified our choice of a PCQ-based network.

Comment 1.2 – Paper contributions are typically three points. The main theme must be highlighted. Since the title and abstract of this paper focus on quantum models, classical preprocessing methods such as diffusion models and SKULLNET should not be given excessive attention, as this obscures the main focus.

Response:

Thank you for pointing this out. In the revised manuscript, we completely removed any mention of segmentation and diffusion models from the "Introduction" and "Methods" sections, as they are only part of the preprocessing step. However, we briefly discussed them in the "Experiments" section because the segmentation model is necessary for dataset variation, and the diffusion model is used to address class imbalance.

Comment 1.3 – The main theme of the main text is not prominent, and there are numerous redundant paragraphs, causing the core method to be completely overshadowed by preprocessing methods. I had to spend a significant amount of time passively accepting basic theories like diffusion models that I already knew. If you want to propose a complete system, you should give it a separate name, such as an AD detection system based on XXX, to encompass all the content. However, the title of this paper is CQ-CNN, so you only need to focus on introducing the novelty and effectiveness of this method. It is recommended to keep the paper length around 14 pages.

Response:

Thank you for the comment. We acknowledge this valuable feedback from the reviewer and have rewritten a significant portion of our manuscript. We removed over four pages of redundant content. Since the paper title, CQ-CNN, suggests a hybrid classical-quantum neural network, our revised manuscript primarily focuses on this topic. Moreover, the manuscript discusses in detail the development of a 3D-to-2D conversion framework, which is also highlighted in the "Abstract," "Introduction," and the title.

Comment 1.4 – For existing methods and models, please do not draw diagrams; simply cite the references. When describing others' methods, keep the description concise and summarise them in a short paragraph.

Response:

Thank you for your valuable suggestion. In response, we have removed all diagrams related to segmentation and diffusion models, as these are established methods. We briefly discussed them in the "Experiments" section. This discussion is limited to about five sentences and includes citations of previous research that utilized these methods.

Comment 1.5 – The experimental model parameter configuration is rather rough. Since this paper involves too many models (including a large number of preprocessing methods, which ultimately undermine the status of the quantum method), detailed configuration parameters such as the number of convolutional kernels and layers in the convolutional neural network are not reflected. It is strongly recommended to list a more detailed table for explanation to facilitate readers' configuration and reproduction.

Response:

Thank you for your valuable comment. In response, we have rewritten the "Experiments" section and briefly included the segmentation and diffusion model training in the preprocessing subsection. We have combined the separate model training configurations into a single table (Table 2). Furthermore, we have introduced a new table (Table 1), which presents a detailed, layer-by-layer configuration of the proposed CQ-CNN model, including the number of convolutional kernels, layers, stride, padding, and the total parameter count.

Comment 1.6 – I am contemplating a core question: Are the superior experimental results you have presented due to extensive preprocessing or the quantum model? Generally, we only perform simple dimensionality reduction on the dataset before directly inputting it into the quantum machine learning model for processing, thereby reducing the impact of various preprocessing methods on the experimental results. Could the results in this paper be due to the superior classical preprocessing? Or, even without the quantum model, the results were already good enough, and adding the quantum model only resulted in a small improvement?

Response:

Thank you for the thoughtful concern. We would like to respectfully clarify that the extensive preprocessing steps are not the primary contributors to the performance of the CQ-CNN model. These steps were included because they are commonly used in prior research involving classical models. Our goal was to ensure that the results were not biased toward any minority class, which often occurs in imbalanced datasets. We also provided justification for using diffusion models over GANs and other data augmentation methods in the "Experiments" section of the revised manuscript. The segmentation model was introduced to create variation in the dataset and to evaluate whether the CQ-CNN performs better on segmented or non-segmented data. Furthermore, many previous studies applied segmentation before classification, which we discussed in detail in the "Results" section, under the "Comparative and computational analysis" subsection.

Regarding the model architecture, CQ-CNN comprises only 13.7K classical parameters alongside the quantum parameters. This is significantly fewer than what is typical in modern classical CNNs. The low parameter count is intentional, allowing us to better isolate and assess the contribution of the PQC. Therefore, the performance of the CQ-CNN model in our results is not attributable to classical preprocessing or the classical model capacity alone, but rather to the combined effectiveness of the hybrid quantum-classical architecture.

Comment 1.7 – Please use the method of controlling variables to ensure fairness in the comparison. Consider this: if the parameter size of ALEXNET were also close to 13K, would its performance be higher than 0.92?

Response:

Thank you for your valuable suggestion. We acknowledge your suggestion regarding controlling variables in ALEXNET to enable a fairer comparison in terms of parameter efficiency. However, in our revised manuscript, the primary objective is to evaluate whether the proposed hybrid classical-quantum model performs effectively on the AD detection task, particularly in a dataset where class images are highly similar. We also aim to examine how increased task complexity affects the performance of classical-quantum networks, and whether our model achieves results comparable to those reported in benchmark studies using more distinct class distributions. The parameter efficiency analysis is presented as a supplementary comparison, where we evaluate our converged CQ-CNN model alongside existing models such as ALEXNET to provide insight into its relative positioning.

Reviewer 2

Comment 2.1 – The technical novelty of the proposed CQ-CNN architecture is not sufficiently clear. Although the combination of classical CNN layers with parameterized quantum circuits (PQCs) is conceptually relevant, similar hybrid models have already been explored in recent literature, including quantum support vector machines and hybrid quantum-classical classifiers. The manuscript does not convincingly demonstrate what new insights or architectural innovations it contributes beyond prior work, especially given the relatively simple PQC configuration (2 or 3 qubits) and standard encoding (ZZFeatureMap) applied.

Response:

We thank the reviewer for the insightful comment. In response, we have rewritten the majority of the manuscript and clearly restated the motivation behind our research. While prior studies have explored hybrid quantum-classical models, their evaluations have mostly been limited to benchmark datasets such as MNIST or CIFAR-10, where class separability is relatively easier. Our work aims to investigate whether these models also perform well on fine-grained image classification tasks like AD detection, where images from different classes are highly similar. We also discussed previous studies on architectures like quantum support vector machines and quanvolutional neural networks. Although these networks exist, most prior work on image classification benchmarks has focused on PQC-based hybrid classical-quantum architectures, which is why we chose to use this type of architecture in our study. Furthermore, since we did not have access to quantum hardware and relied on CPU-based simulations, using more than 3 qubits was computationally impractical due to the exponential increase in time complexity. In addition, we used the ZZFeatureMap because it provides angle encoding with entanglement, which is computationally efficient and well-suited for small-qubit simulations.

Comment 2.2 – The performance comparisons lack depth. The study compares the CQ-CNN only to a few classical models with matched parameter counts, but fails to benchmark against more competitive lightweight deep learning models or state-of-the-art AD classification frameworks. Moreover, no statistical tests are provided to assess the significance of the reported improvements. The claim of quantum advantage based on reduced epoch count is weak without clearer control experiments to rule out confounding variables such as optimizer settings, dataset variance, or model initialization.

Response:

We appreciate the reviewer's insightful feedback. However, we would like to respectfully clarify that most prior studies on AD classification have predominantly utilized heavyweight classical CNNs, often comprising tens of millions of parameters. Even the lightweight CNN variants typically contain around a million parameters, and methods like knowledge distillation still require training a large-capacity teacher model. In our study, we did not limit comparisons to parameter counts alone. We also evaluated models under additional constraints such as CPU-only simulations, segmentation dependency, model size, and GPU requirements. These factors are now clarified in the revised manuscript to provide a more comprehensive and practical perspective on model deployment. We now also emphasize that further improvements in quantum optimization techniques are necessary to make current-generation hybrid classical-quantum models practical for real-world medical imaging applications such as automatic AD detection.
In addition, we have expanded our analysis to address the limitations of hybrid classical-quantum architectures. The revised manuscript includes new control experiments demonstrating specific

Comment 2.3 – The experiments rely heavily on a small binary subset of the OASIS-2 dataset (non-demented vs. moderate demented), which reduces the complexity and clinical relevance of the classification task. The exclusion of mild and very mild cases, which are clinically important for early diagnosis, undermines the model's practical utility. Additionally, the segmentation model (SkullNet) and the synthetic augmentation via diffusion models are not sufficiently validated—there is no qualitative or clinical expert evaluation of the generated samples, nor any ablation study to quantify the impact of segmentation or augmentation on final classification performance.

Response:
We thank the reviewer for the thoughtful comment. In the revised manuscript, the "Discussion and Limitations" section explains our rationale for conducting binary classification and excluding the mild and very mild cases in the OASIS-2 dataset. Although we attempted a multi-class setup, the increased task complexity caused the hybrid classical-quantum models to struggle with convergence, which contrasts with prior studies on benchmark datasets using similar architectures. We have elaborated on this issue in the manuscript. We acknowledge that the excluded classes are critical for early diagnosis, and in light of this, we have removed all claims related to the practical deployment of our architecture without significant improvements in gradient optimization techniques.
Since the segmentation model and the generative diffusion model are part of our preprocessing pipeline, we have removed their detailed descriptions from the Methods" section. They are now briefly discussed in the Experiments" section, where we also reference prior studies to justify our choice of architectures. Based on our review of related work with classical

models, generated synthetic data and segmented images are typically not clinically validated when the primary research focus is on architectural performance rather than data preparation. If our work had focused on dataset creation, we would have pursued such clinical validations. In addition, the Results" section includes a comparison between skull-stripped (segmented) and non-skull-stripped (non-segmented) datasets. Our Ablation Study" section also provides a comparative analysis with previous studies using skull-stripping techniques.

Comment 2.4 – The manuscript makes claims about low resource requirements and lightweight deployment, yet no real-world deployment tests (e.g., on actual quantum hardware or edge devices) are reported. Given the current limitations of NISQ-era quantum simulators, the feasibility of using such quantum circuits in real clinical settings remains speculative.

Response:

Thank you for the valuable feedback. We acknowledge the concern, and in the revised manuscript, we have removed all claims regarding practical and lightweight deployment. In addition, we have expanded the "Discussion and Limitations" section to explicitly address this concern. Specifically, we clarify that the high cost and limited availability of NISQ-era quantum devices prevented us from testing our models on real quantum hardware.

Comment 2.5 – The manuscript does not sufficiently engage with the latest research in the field. Recent works such as those recently published—specifically, DOI: 10.1002/advs.202506213 and DOI: 10.1002/advs.202411573—hi[illegible] more advanced and targeted approaches to hybrid quantum learning and should be discussed in the context of this work.

Response:

We thank the reviewer for highlighting these important recent works. We have incorporated a discussion of the quantum kernel learning methods in the "Discussion and limitations" section and incorporated the suggested references. These methods, which feature a self-adaptive quantum kernel PCA for dimensionality reduction and a quantum kernel-aligned regressor for modeling small, high-dimensional datasets, offer promising potential to improve gradient optimization and model convergence. We suggest that evaluating these approaches could help address the optimization challenges present in our current framework.

Reviewer 3

Comment 3.1 – While the manuscript claims quantum advantage, the evidence remains circumstantial and limited to simulations. No real quantum hardware results are presented, and the simulations are small-scale (2–3 qubits), limiting generalizability. Furthermore, claims of superior convergence require rigorous statistical testing (e.g., confidence intervals, ANOVA) to establish significance beyond classical baselines.

Response:

We sincerely thank the reviewer for this insightful observation. In the revised manuscript, we have clarified that our primary objective is to evaluate the performance of current-generation hy- brid classical-quantum convolutional neural networks (CNNs) on classification tasks characterized by high inter-class similarity. Accordingly, we have moderated our prior claims regarding quantum ad- vantage, explicitly acknowledging that all experiments were conducted using CPU-based simulations rather than actual quantum hardware. However, we maintain that parameter efficiency represents a meaningful quantum advantage in our work. Despite operating with only 2–3 qubits, our hybrid classical-quantum models achieve comparable performance to significantly larger classical models. We have also detailed the rationale for limiting simulations to 2–3 qubits: scaling the number of qubits leads to exponential increases in computational cost and training time, as well as increased gradient instability, without guaranteed improvements in performance.

Regarding the reviewer's point about convergence and statistical significance, we incorporated additional control experiments using the MNIST dataset. Specifically, we conducted 30 additional experiments to rigorously evaluate training stability and feature separability. These results are shown in Figure 11, where the top row presents t-SNE visualizations showing MNIST features forming clear, separate clusters, while OASIS-2 MRI features overlap between non-dementia and moderate dementia cases. The bottom row shows training accuracy curves over five independent runs, annotated with ANOVA F-statistics and corresponding p-values, which assess variability and convergence stability.

Finally, we have expanded the "Discussion and limitations" section to explicitly address con- straints related to the limited availability and high costs of real quantum hardware, which prevented empirical validation on physical quantum devices. We hope these clarifications and revisions suffi- ciently address the reviewer's concerns and improve the rigor and transparency of our claims.

Comment 3.2 – The exclusion of "very mild" and "mild" dementia classes significantly weakens the clinical relevance of the binary classifier. These classes are critical for early detection. The reasoning for this exclusion is inadequately justified, and the impact of this simplification is not discussed in the limitations. Consider ordinal or multi-class classification, or explicitly discuss the limitations of binary simplification.

Response:

Thank you for the valuable suggestion. In the revised manuscript, we explicitly address the reviewer's concern in the "Discussion and Limitations" section. We acknowledge that including the "very mild" and "mild" dementia classes is important for early diagnosis. However, current-generation hybrid classical-quantum architectures are not yet mature enough to effectively handle such complex multi-class classification tasks. We have statistically demonstrated the limitations of these models, including gradient instability and convergence challenges, and discussed these issues in detail. As a result, we have removed any claims that our model or current hybrid classical-quantum models are ready for clinical deployment. Instead, we emphasize the need for further advances in quantum optimization techniques to make these models practical for real-world medical imaging applications such as automatic AD detection.

Comment 3.3 – The manuscript acknowledges convergence instability due to quantum gradient vanishing/exploding problems, especially with more qubits. However, there is no exploration of advanced techniques like layer-wise learning rates, gradient clipping, or parameter-shift rule variants that could address this issue. Integrate more robust quantum optimization strategies or provide ablation studies that test the effect of different PQC structures or initialization methods.

Response:

We thank the reviewer for the thoughtful and constructive comment regarding the convergence instability in the quantum component of our hybrid classical-quantum CNN (CQ-CNN) model. As outlined in the "Ablation study" section, we experiment extensively with various optimizers, including SGD, L-BFGS, RMSprop, Adagrad, and Adam. Among these, only Adam enables convergence, as its adaptive learning rate and momentum mechanisms help manage the unstable gradients in PQC. We also increase the classical model capacity by adding larger convolutional filters, but this does not resolve the stagnation, confirming that the core issue arises from the quantum layer, particularly due to the high inter-class similarity present in the OASIS-2 dataset.

We acknowledge that, while these investigations provide valuable insights, we do not integrate the advanced optimization strategies suggested by the reviewer in this study. One of

the main constraints is the computational overhead and framework limitations of simulating quantum circuits with Qiskit, which limits the feasibility of systematically testing these techniques within the current scope. However, we recognize the potential of these methods to address quantum gradient instability and improve the convergence behavior of hybrid models.

In response to this important point, we update the "Discussion and limitations" section to explicitly include these optimization strategies. We discuss how gradient clipping, noise-aware gradient regularization, improved PQC parameter initialization, and more efficient gradient estimation methods such as parameter-shift rule variants may help address optimization challenges in CQ-CNN training and form key directions for our future research.

Reviewer 4

Comment 4.1 – The paper's emphasis on parameter efficiency, citing a total of only 13K parameters, warrants a more detailed analysis. The manuscript does not provide a parameter breakdown between the classical CNN backbone and the Parameterized Quantum Circuit (PQC). Without this information, it is difficult to assess to what extent this efficiency is attributable to the quantum component versus a potentially minimalistic design of the classical network itself. Consequently, comparing the model's total parameter count to that of large-scale classical models like AlexNet (60M parameters) may overstate the unique contribution of the PQC to the model's compactness.

Response:

We thank the reviewer for this insightful observation. In response, we have revised the manuscript to include a detailed parameter breakdown between the classical CNN backbone and the Parameterized Quantum Circuit (PQC), as shown in the updated Table 1. Specifically, we now explicitly report the parameter counts for each classical layer, the PQC, and the associated fully connected layers leading to the PQC. This breakdown clarifies that the classical CNN backbone constitutes 13,718 parameters, while the number of parameters contributed by the PQC is denoted as Q, and the intermediate fully connected layer preceding the PQC contributes $\omega_1 = (4 \text{ x } \omega) + \omega$.

We also acknowledge the reviewer's point regarding the comparison with large-scale classical models such as AlexNet. In our study, we did not limit comparisons to parameter counts alone. We evaluated models under additional constraints including CPU-only simulations, segmentation dependency, and model size. Consequently, we have removed the claim that our model outright outperforms AlexNet from the abstract and discussion, and instead included this comparison in the "Comparative and computational analysis" subsection. Nonetheless, our best converged model performs on par with state-of-the-art methods despite significant computational constraints, and this is now explicitly stated in the "Abstract" and "Conclusion".

Comment 4.2 – The work encompasses a complete and complex pipeline. This makes the paper's primary contribution unclear. Is the core innovation the novel CQ-CNN architecture itself, or is it the entire end-to-end clinical workflow, which integrates segmentation and diffusion models with a quantum classifier for the first time in this context? Components such as using U-Net for segmentation or diffusion models for data generation are not, in themselves, new concepts. The authors should more precisely frame their novelty around the integration and application of this full stack within a hybrid quantum computing context.

Response:

We sincerely thank the reviewer for highlighting this important point. In response, we have revised the manuscript to more clearly emphasize the core contribution of our work. Specifically, we have removed the extensive descriptions of the segmentation model and the generative diffusion model from the "Methods" section, as these are standard preprocessing steps and do not constitute novel contributions. Instead, these components are now briefly discussed only in the "Experiments" section, where we clarify that the segmentation model was employed to introduce dataset variation, while the diffusion model was used to address class imbalance.

Furthermore, we have rewritten the "Introduction" section to eliminate redundancy and refocus the narrative on the primary objective of our research: evaluating the effectiveness of a hybrid classical-quantum neural network (CQ-CNN) for AD detection, particularly in scenarios where class images exhibit low inter-class variation. We have also strengthened the positioning of our work by reviewing prior studies on hybrid classical-quantum models, particularly those implemented on quantum hardware, and provided justification for adopting a PQC-based classifier in our architecture.

Comment 4.3 – The paper claims SOTA performance by comparing the CQ-CNN to several existing models in Table 5. However, a closer look reveals that many of the compared models were evaluated on different datasets (e.g., ADNI, OASIS-3), which makes a direct performance comparison challenging. To ensure fairness and accuracy, the authors should refine their claim to be more specific, for instance, by stating that they have achieved SOTA performance "on the OASIS-2 dataset for the specified binary classification task."

Response:

We appreciate the reviewer's observation and agree that direct accuracy comparisons across different datasets can be limiting. In our revised manuscript, the primary goal is not to claim absolute SOTA across classical models trained on OASIS-2, but rather to position

our CQ-CNN models within the broader context of AD detection. Our focus has been on highlighting parameter efficiency, computational constraints, and segmentation strategies, in addition to overall performance. Since quantum-enhanced models currently lack optimized GPU support and rely on CPU-based simulations, achieving competitive performance under these hardware constraints is a meaningful contribution.
It is also important to note that most of the classical models we compared against were trained for binary classification tasks, which is consistent with our experimental setup. In response to the reviewer's concern, we have revised the manuscript to avoid any statements suggesting that our models definitively outperform classical SOTA models. Instead, we emphasize that our contributions demonstrate competitive performance within the given computational constraints.

Comment 4.4 – The paper admits that the CQ-CNN models suffer from convergence issues, stemming from gradient failure in the quantum component, which necessitates "multiple re-runs of experiments before achieving satisfactory performance". If a model frequently fails to converge, can its occasional rapid convergence be robustly framed as an "advantage"? The authors should discuss this trade-off between potential speed and practical instability more deeply, and quantify this instability to allow for a proper assessment of the model's experimental reproducibility and stability.

Response:

We thank the reviewer for raising this important point. To address concerns about the frequency of convergence failures and the stability of our CQ-CNN models, we conducted additional control experiments using the MNIST dataset. In these experiments, we ran 30 independent training trials to rigorously assess training stability. The results demonstrated consistent and reliable convergence on MNIST, indicating that the convergence issues observed with the OASIS-2 dataset arise not from inherent architectural flaws but from the increased complexity and low inter-class variation present in the AD classification task.
In the revised manuscript, we have expanded the "Discussion and Limitations" section to explore this trade-off more thoroughly. We also quantify the instability observed in our experiments and emphasize that while occasional rapid convergence offers advantages in parameter efficiency, it is accompanied by practical challenges related to stability, limited availability of quantum hardware, and the constraints of current simulation frameworks. Furthermore, we propose several future research directions, including advanced quantum optimization techniques and architectural improvements, to mitigate these issues.
We have also removed any bold claims about our model outperforming classical SOTA models or being readily deployable. Instead, we emphasize the need for further advancements in quantum optimization to make these models practical for real-world scenarios. Nevertheless, we highlight the competitive performance of our model, as the converged CQ-CNN achieved strong results under significant computational constraints.

Comment 4.5 – The paper includes a valuable ablation study by comparing results with and without skull-stripping. However, the necessity of another key, complex component—the probabilistic diffusion model—is not experimentally justified. The authors argue it is superior to traditional augmentation, but this is not demonstrated. A crucial ablation study comparing the results of the diffusion model against simpler augmentation techniques (e.g., rotation, flipping) and a no-augmentation baseline would be essential to justify the inclusion of this computationally intensive step.

Response:

Thank you for the valuable suggestion. We would like to respectfully clarify that following many other comments, in the revised manuscript, we removed the detailed descriptions of the segmentation and diffusion models from the "Methods" section to reduce redundancy. Instead, these steps now appear only briefly in the "Experiments" section as standard preprocessing rather than core novelties.

Regarding the justification for using the diffusion model, we clarified that simpler augmentation techniques such as rotations and flipping are not feasible because preserving the spatial orientation of MRI scans is critical for accurate AD classification. In addition, synthetic data generators such as generative adversarial networks (GANs) typically require large amounts of training data, which is not available for the minority class in our dataset. Therefore, we chose diffusion-based synthetic data generation as a more suitable approach to address class imbalance while maintaining anatomical consistency.
