# Supplementary material for: CQ-CNN: A lightweight hybrid classical–quantum convolutional neural network for Alzheimer’s disease detection using 3D structural brain MRI
Source: PLoS One. 2025 Sep 22;20(9):e0331870. doi: 10.1371/journal.pone.0331870 (PMC12453261; doi:10.1371/journal.pone.0331870)
Supplement: Attachment — Submitted filename: Response_to_Reviewers_auresp_2.pdf [file pone.0331870.s002.pdf]

August 19, 2025

Editor-in-Chief
PLOS ONE

Dear Editor-In-Chief,

We want to express our sincere gratitude for granting us the opportunity to submit the revised manuscript (Ref. No.: PONE-D-25-31817R1) titled "CQ-CNN: A lightweight hybrid classical–quantum convolutional neural network for Alzheimer's disease detection using 3D structural brain MRI" to PLOS ONE. We greatly value the time and effort you and the reviewers invested in our manuscript, which has significantly improved its quality and rigor.

Your feedback and the reviewers' suggestions were truly invaluable. We are pleased to inform you that we have carefully addressed all the reviewers' questions and comments, making the necessary revisions to the manuscript.

Our responses to the reviewers' comments are included in the following pages, with page numbers referring to the revised manuscript that incorporates tracked changes. As the corresponding author, I confirm that all authors have reviewed the manuscript, agreed to the changes, and consented to the submission of this revised version.

We genuinely appreciate your consideration of our manuscript.
Best Regards,

Dr. M. R. C. Mahdy (Corresponding Author)
Professor
Department of Electrical and Computer Engineering
North South University, Dhaka, Bangladesh
E-mail: mahdy.chowdhury@northsouth.edu

Response to The Editorial Board

Academic Editor's Comments

1. Reviewers raised some new issues, along with concerns about poorly addressed previous comments. Please revise your manuscript according to the comments below, paying particular attention to avoid directly adopting any contexts from LLM. Please DO proofread and make all expressions tight and clear. Thanks.

   Response:

   We sincerely thank the Academic Editor for this important reminder. We confirm that no part of our manuscript was drafted using LLM or GPT tools; all text and analyses are original contributions of the authors. To ensure full compliance, we have carefully proofread the entire manuscript multiple times, with special attention to clarity, conciseness, and consistency. Expressions have been tightened, verbose passages reduced, and terminology unified throughout. These revisions address both previously raised and newly identified concerns, ensuring the manuscript now meets the standards of PLOS ONE in terms of originality, precision, and readability.

Note to The Reviewers

1. All modified portions in the revised manuscript are highlighted in yellow colored text.
2. In answering the comments, we have used the following format throughout this response letter. We first state the comments made by the respective reviewer, followed by our response.

[**Tribute**] We would like to express our deepest appreciation to the anonymous reviewers for their extremely insightful comments that greatly enhanced the quality of the paper. We have made every effort to implement the suggestions, demonstrating the effectiveness of our work.

Response to The Reviewers

Responses to the review comments are illustrated below:

Reviewer 1

Comment – Most importantly, the authors failed to directly address all of my questions, especially the last two, without providing a reasonable explanation. Second, the citation standards are not corrected. The paper is too long, and the text is overly verbose. In short, it fails to achieve the concise and straightforward approach expected of an academic paper. Third, the paper uses numerous terms interchangeably, without any consistency. This is a basic error, but it is likely a common one for GPT. For example, PQC and parameterized quantum circuit appear interchangeably. What is the meaning of the abbreviation PQC? Based on the above, I believe this paper is full of flaws and redundant content.

Response: We thank the reviewer for their feedback and have carefully revised the manuscript to address all concerns. We also confirm that no content in the manuscript was generated by any LLM or GPT tool; all text and analysis are originals. The repetitive and inconsistent use of abbreviations, such as PQC, resulted from multiple authors contributing to separate sections and unfortunately escaped our initial proofreading. These issues have now been corrected, and the entire manuscript has been carefully proofread for clarity and consistency.

i. Directly addressing the last two comments:

Comment 6: *I am contemplating a core question: Are the superior experimental results you have presented due to extensive preprocessing or the quantum model? Generally, we only perform simple dimensionality reduction on the dataset before directly inputting it into the quantum machine learning model for processing, thereby reducing the impact of various preprocessing methods on the experimental results. Could the results in this paper be due to the superior classical preprocessing? Or, even without the quantum model, the results were already good enough, and adding the quantum model only resulted in a small improvement?*

Reply: To address the reviewer's concern, we conducted additional controlled experiments to disentangle the effects of preprocessing from the contribution of the quantum model. We introduced the γ_8-classical model, a purely classical CNN with the same number of parameters as our α_8-2-qubit and β_8-3-qubit CQ-CNN models. The γ_8-classical model achieved an accuracy of 0.9197 on OASIS-2. In comparison, the α_8-2-qubit and β_8-3-qubit models improved accuracy by 1.63% and 5.67%, respectively. These results indicate that even when parameter count and preprocessing are held constant, the inclusion of quantum layers provides measurable performance gains. Thus, the observed improvements cannot be solely attributed to preprocessing, and the PQC layers meaningfully enhance the model's representational capacity. While preprocessing contributes to baseline performance, the quantum components are responsible for the additional improvement and are not redundant.

Comment 7: *Please use the method of controlling variables to ensure fairness in the comparison. Consider this: if the parameter size of ALEXNET were also close to 13K, would its performance be higher than 0.92?*

Reply: As suggested by the reviewer, we performed a controlled-variable experiment to ensure a fair comparison with classical baselines. We implemented a reduced-scale AlexNet†, constrained to a parameter count comparable to our CQ-CNN models (13.7K parameters). This reduced-scale AlexNet† achieved an accuracy of 0.8554, which is 8.5% lower than the α_8-2-qubit model and 12.3% lower than the β_8-3-qubit model. This result demonstrates that even when classical models are scaled to match the parameter count of our CQ-CNN, their performance remains significantly lower. Therefore, the observed performance improvements are primarily attributable to the inclusion of quantum layers rather than parameter size alone, validating the contribution of the PQC in enhancing model capacity.

ii. Citation standards: We have checked the formatting of the references, and it complies with the journal's style.

iii. Terminology consistency: All terms are now used consistently. The abbreviation PQC is explicitly defined as Parameterized Quantum Circuit at first mention in the Introduction, and this terminology is consistently applied throughout the manuscript. Its abbreviation is also mentioned in the Abstract, which is separate from the main text, to clearly convey the key contribution of the quantum component upfront.

iv. Conciseness, verbosity and manuscript length: We acknowledge the reviewer's suggestion to shorten the manuscript. Redundant paragraphs, particularly in the "Introduction" and "Methods" sections, were already removed in previous revisions. Although the overall length is now approximately 24 pages, this is due to the addition of new experiments, ablation studies, extended analyses of convergence and stability, and a more thorough discussion of limitations, elements requested by other reviewers to strengthen the rigor and reproducibility of the work. All new material has been presented concisely to maintain clarity.

v. Incorporation of Comments 1–5: We believe that Comments 1–5 have already been fully incorporated in the manuscript through:

- Rewritten "Introduction" and "Methods" sections, with an emphasis on the CQ-CNN architecture rather than preprocessing (Comments 1 & 3),
- Detailed model parameter tables for reproducibility (Comment 5),
- Removal of segmentation and diffusion details from Introduction and Methods, with their usage only cited in Experiments (Comment 2 & 4),
- Clear, consistent terminology throughout (New Comment).

We greatly appreciate the reviewer's constructive feedback, which has helped improve the manuscript. We believe that the additional experiments and careful revisions fully

address all concerns, clarify the manuscript's main contributions, and demonstrate the significance of the quantum-enhanced CQ-CNN model.

Reviewer 2

Comment – None

Response:
We express our thanks and appreciation to the reviewer for taking the time for a careful reading of our manuscript and for the very insightful comments.

Reviewer 3

Comment – The authors have satisfactorily modified their manuscript according to my previous criticisms. Therefore, I recommend the publication of this manuscript.

Response:

We express our thanks and appreciation to the reviewer for taking the time for a careful reading of our manuscript and for the very insightful comments.

Reviewer 4

Comment 4.1 – Regarding the SOTA claims (Comment 4.3): In the revised manuscript, the authors have appropriately shifted their focus from claiming definitive state-of-the-art (SOTA) performance to demonstrating that their model achieves "comparable performance" under significant computational constraints . To fully substantiate this revised claim, the paper would be significantly strengthened by including a direct, rigorously controlled baseline. This would involve training a classical CNN with a similar parameter count (e.g., replacing the PQC with classical dense layers) under the exact same experimental conditions (same data splits, resolution, and augmentation). Such an experiment would provide the necessary fair comparison point to empirically validate the model's performance and truly assess the PQC's contribution.

Response:

We thank the reviewer for emphasizing the importance of a fair comparison to substantiate our claims. To address this, we included a direct baseline using a fully classical CNN with the same parameter count as our PQC-based models. Specifically, the γ_8-classical model, matching the parameter count of the α_8-2-qubit and β_8-3-qubit models, was trained under identical experimental conditions (same data splits, resolution, and augmentation). The results show that the γ_8 classical model achieved an accuracy of 0.9197. In comparison, the PQC-embedded α_8-2-qubit model achieved an accuracy of 0.9350, an improvement of 1.63%, and the β_8-3-qubit model achieved an accuracy of 0.9750, an additional improvement of 5.67%. These results demonstrate that, even with an identical parameter count, the inclusion of quantum layers enhances representational capacity and provides measurable performance gains. Thus, our revised claim of achieving "comparable performance" is now supported by a rigorous, controlled baseline.

Comment 4.2 – Regarding the convergence instability (Comment 4.4): The new control experiment presented in Figure 11 provides a compelling empirical finding that the model's instability is data-dependent. To elevate the impact of this finding, the manuscript should now connect this observation to a deeper theoretical discussion of known challenges in Quantum Machine Learning (QML). For instance, the discussion could posit whether the observed "gradient failure" is a manifestation of barren plateaus, which may be more likely to occur when the quantum feature map encodes highly entangled states from a dataset with subtle feature differences. Integrating this theoretical analysis would provide a more profound explanation for the observed behavior and strengthen the core findings of the paper.

Response:

We appreciate the reviewer's suggestion to provide a deeper theoretical context for the observed data-dependent instability. In the revised manuscript, we discuss this phenomenon in the context of barren plateaus, where gradients can vanish exponentially with the number of qubits or the expressibility of the quantum circuit, making optimization increasingly difficult (lines 496–479, revised manuscript).
